# Metal Chalcogenides with Heterostructures for High‐Performance Rechargeable Batteries

**DOI:** 10.1002/smsc.202100012

**Published:** 2021-06-04

**Authors:** Yu Li, Feng Wu, Ji Qian, Minghao Zhang, Yanxian Yuan, Ying Bai, Chuan Wu

**Affiliations:** ^1^ Beijing Key Laboratory of Environmental Science and Engineering, School of Materials Science and Engineering Beijing Institute of Technology Beijing 100081 P. R. China; ^2^ Collaborative Innovation Center of Electric Vehicles in Beijing Beijing 100081 P. R. China

**Keywords:** alkali metal−ion batteries, heterostructures, lithium−sulfur batteries, metal−air batteries, metal selenides, metal sulfides, multivalent ion batteries

## Abstract

Heterostructures exhibit intriguing and significant properties for functional material applications, such as photosensing devices, semiconductor materials, and supercapacitors. Rechargeable batteries as typical energy‐storage devices have drawn widespread attention in the past several decades, on account of high energy density, being low‐cost, and ecofriendly. Preparing superior active materials is the critical technology to ameliorate the electrochemical performance of batteries. In recent years, the concept of constructing heterostructures for the application of electrode materials has been considered as a promising design approach. Among all the electrode materials, metal chalcogenides (MCs) have presented excellent properties due to their high theoretical capacity based on multielectron reaction. Herein, the progress on MCs with heterostructures is summarized in terms of various material species and their specific application for several typical battery systems. Finally, possible challenges and comprehensive perspectives are given to provide an instructive direction for the thoughtful design strategies of heterostructures and the development of MCs for next‐generations rechargeable batteries.

## Graphical abstract

Constructing heterostructures for advanced electrode materials have been considered as a potential design strategy. Metal chalcogenides (MCs) have presented excellent electrochemical performances due to their high theoretical capacity based on multielectron reaction. Herein, the progress on MCs with heterostructures is summarized in terms of various material species and their specific application for several typical battery systems.

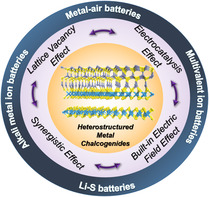

## 1 Introduction

The high‐speed development of economy and the enhanced awareness of environmental protection are providing huge opportunities and challenges for the electrochemical energy storage (EES) field. Especially, rechargeable batteries as typical energy‐storage devices have attracted much attentions in the past few decades, due to their superiorities like high energy density, being low‐cost, and ecofriendly.^[^ ^1^ ^]^ Nevertheless, they must face a series of issues caused by the ever‐growing demands for higher electrochemical performance and longer cycle life. As discussed, batteries’ performances mainly depend on the active materials including cathodes and anodes.^[^ ^2^ ^]^ Researchers have made tremendous efforts to fabricate excellent active materials through various synthesis methods and ingenious processing technologies, like coating conductive materials,^[^ ^3^ ^]^ doping heteroatoms,^[^ ^4^ ^]^ designing specific morphologies (nanostructures,^[^ ^5^ ^]^ hollow structures,^[^ ^6^ ^]^ core−shell structures,^[^ ^7^ ^]^ hierarchical structures,^[^ ^8^ ^]^ etc.), etc. Therefore, constructing heterostructures composed of two or more different components has been recently emerged and wildly studied, due to the fact that they may amalgamate the advantages of single components and even endow new functions to further improve their properties.^[^ ^9^ ^]^

To further elevate the energy density of rechargeable battery systems, considerable attention has been paid to exploring active materials based on the chemistry of multielectron reactions, which are expected to provide a higher theoretical capacity. Among these multielectron reaction materials, metal chalcogenides (MCs) have become research hotspots and show great prospects for application due to their layer‐dependent bandgap, which can offer excellent electronic and mechanical performance.^[^ ^10^ ^]^ MCs can be defined as a generalized abbreviation of MCs, where M is a metal atom (Fe, Co, W, Mo, Sn, and so on) and C is a chalcogen atom (S, Se, and Te). They usually have an open structure with a weak interlayer Van der Waals force and may contribute to fast intercalation/deintercalation for ions (Li^+^, Na^+^).^[^ ^11^ ^]^ In addition, the electrochemical reaction mechanisms of MCs are mainly based on the conversion reaction or alloying reaction, which can achieve higher reversible capacity and make them more outstanding compared with other electrode active materials. However, the electrochemical performances of MC electrodes for batteries are still severely restricted by many problems, like their intrinsic electronic conductivity, extensive aggregation, and unstable bulk structure (terrible volume expansion) during the charge/discharge process.

In recent years, constructing MCs with heterostructures, namely, hybridization of MCs with other analogous functional materials, is becoming an interesting strategy to synthesize a new‐type composite, which can solve the aforementioned limitations. Several published review articles mainly focused on the synthesis and application for various MCs. Until now, research efforts on the construction of heterostructures among MCs have not been systemically investigated. In this Review, we first give a brief introduction on the design, preparation, and significance for MCs with heterostructures and then comprehensively summarize the most recent progresses of MCs with heterostructures in the field of rechargeable batteries and expect to provide outlooks and perspectives of material architecture for next‐generation energy‐storage systems.

## 2 Design, Preparation, and Significance for MCs with Heterostructures for Rechargeable Batteries

Rechargeable batteries such as lithium‐ion batteries (LIBs) are progressively becoming the basic energy devices in our daily life and production on account of their long‐term cycle life and satisfactory energy density. To reach higher standards for energy density and ensure sustainable development of batteries, some new‐type rechargeable batteries have successively appeared, including sodium‐ion battery (SIB), potassium‐ion battery (KIB), lithium−sulfur (Li−S) battery, aluminum‐ion battery, and so on. As discussed, electrode material is the key component of battery systems, which is in direct relation to the overall battery's performance. The rational design for electrode can significantly enhance the structural stability and elevate the specific capacity.

The design concept of heterostructured MC fabrication could start from the several typical hybrids, including same metal with dissimilar chalcogenides, bimetal chalcogenides, two or more different MCs, and MCs combined with carbonaceous material or other functional materials. Generally, there are physicochemical similarities among each single component, which may make them effectively integrated and present great potential for various applications.

So far, many synthesis methods have been developed to prepare heterostructured MCs with particular characteristics, such as various dimensional materials (1D, 2D, or 3D), unique structural morphology, and different microsizes. The most common approach is the hydrothermal or solvothermal method, which possesses many advantages like fast synthesis and easy process. A certain amount of metal salts, S or Se sources, and suitable surfactants are dissolved in aqueous/organic solution to react under strong acid or alkali conditions. The high temperature and pressure should result in the product with complete crystal shape, uniform particle size, and perfect micromorphology. In addition, metal–organic frameworks (MOFs) have received much consideration for the synthesis for MCs and porous carbon when used as precursors. Due to the effective combination of metal ions and organic ligands, heterostructured MCs can be successfully synthesized followed by sulfuration or selenation. The thermal reduction method is also used to fabricate the MCs. For instance, Shi and coworkers^[^ ^12^ ^]^ adopted an in situ carbothermal reduction method to successfully prepare the Co_9_S_8_/CoO heterostructures. In addition, chemical vapor deposition (CVD) is normally operated under a gaseous environment and a high temperature. Compared with the common wet chemistry routes, this method would create relatively tight interfaces among each single component of as‐prepared heterostructured MCs. Sun and coworkers^[^ ^13^ ^]^ used CVD approach to prepare NG/ReSe_2_ hybrids on the 3D Ti_3_C_2_ MXene supports. The achieved NG/ReSe_2_/MXene composites with heterostructures show outstanding performances when used as an advanced anode material for KIBs. On the whole, the synthesis methods to prepare heterostructured MCs mainly include hydrothermal or solvothermal method, MOF‐assistant method, thermal reduction method, CVD method, coprecipitation method, spray pyrolysis method, solid‐state reaction method, and so on.

**Figure 1:**
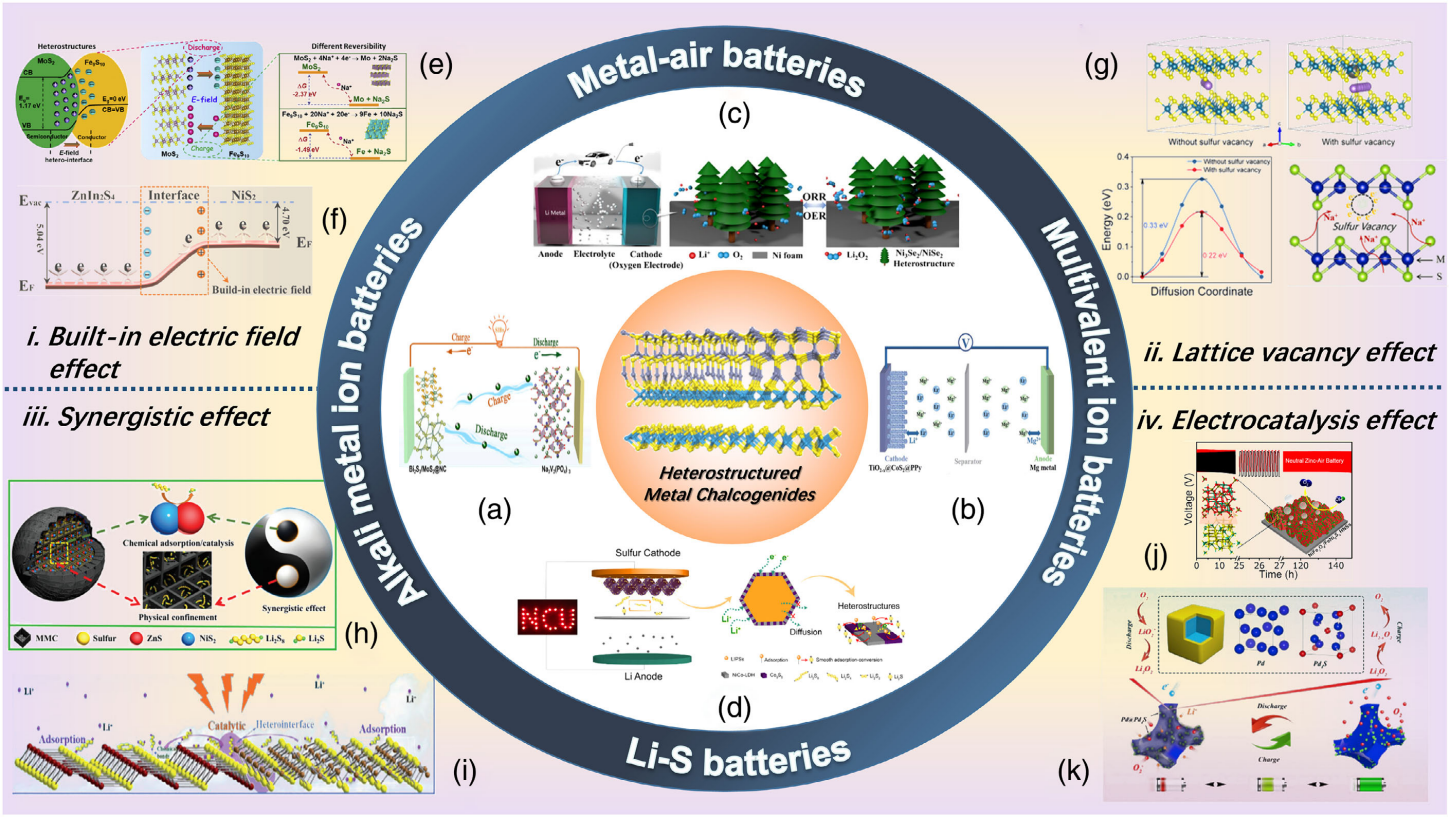
Overview of heterosturctured MCs in rechargeable secondary batteries. a) Reproduced with permission.^[63c]^ Copyright 2020, American Chemical Society. b) Reproduced with permission.^[^ ^95^ ^]^ Copyright 2020, The Royal Society of Chemistry. c) Reproduced with permission.^[78c]^ Copyright 2020, Elsevier B.V. d) Reproduced with permission.^[^ ^72^ ^]^ Copyright 2020, Elsevier B.V. e) Reproduced with permission.^[^ ^64^ ^]^ Copyright 2019, Elsevier B.V. f) Reproduced with permission.^[78a]^ Copyright 2020, American Chemical Society. g) Reproduced with permission.^[^ ^14^ ^]^ Copyright 2020, Wiley‐VCH. h) Reproduced with permission.^[70b]^ Copyright 2020, The Royal Society of Chemistry. i) Reproduced with permission.^[70a]^ Copyright 2020, The Royal Society of Chemistry. j) Reproduced with permission.^[^ ^84^ ^]^ Copyright 2018, American Chemical Society. k) Reproduced with permission.^[^ ^75^ ^]^ Copyright 2020, Elsevier B.V.

The relation between heterostructure and performance should be comprehensively understood. As shown in **Figure** **1**, the main reasons for improving electrochemical performance based on heterostructures are listed as follows. 1) Built‐in Electric Field Effect: The energy bandgap difference between two components (A and B) should induce a stronger electric field at the heterointerface, demonstrating the electron flux from one to another when the heterojunction is formed. Therefore, one becomes an n‐type semiconductor; meanwhile, another turns out to be a p‐type one. Because of the imbalanced charge distribution in the A/B heterostructure, a built‐in electric field (BIEF) at the interface may be induced. The BIEF developed in the heterogeneous interface would push alkali metal ions from A to B or B to A during the electrochemical cycle process, which can lead to excellent ion transport kinetics. 2) Lattice Vacancy Effect: During the synthetic process, it may inevitably form lattice vacancies (sulfur vacancies or selenium vacancies) among heterostructured MCs. For instance, sulfur vacancies nay induce excess electrons around the corresponding metal atoms, being treated as centers for negative charge to effectively attract alkali metal ions and elevate ion diffusion kinetics.^[^ ^14^ ^]^ Lattice vacancies can also act as an electronic charge carrier to significantly reinforce the electrical conductivity. In addition, vacancies may provide extra active sites for redox reactions to enhance the capacitance behavior. In short, lattice vacancy effect will markedly improve ionic and electronic transport kinetics. 3) Synergistic Effect: Synergistic effects are the combined effects of two or more active ingredients making an impact that is more significant than all of them could have shown by themselves. One representative case is the construction MCs with heterostructures for the Li−S batteries. MCs always show high adsorption ability toward polysulfides, and they can promote conversion on account of its catalysis. The well‐designed MC‐based materials with heterostructures will form abundant active interfaces and realize the trapping−diffusion conversion of polysulfides based on the synergistic effect. 4) Electrocatalysis Effect: MCs have been demonstrated to availably catalyze the oxygen evolution reaction (OER) and oxygen reduction reaction (ORR), due to their alterable bandgaps, tunable surface chemistry, and abundant edge and plane active sites. The MCs with heterostructures always possess rich reaction sites and active interfaces for ORR/OER reactions, and it enables the applications of heterostructured MCs as the promising electrocatalysts for metal−air batteries.

In a word, remarkable progress has been made for adopting MCs with heterostructures in many battery systems, showing stupendously improved performance. Here we will mainly review four categories of rechargeable batteries: alkali metal−ion batteries, Li−S batteries, metal−air batteries, and multivalent ion batteries.

## 3 Heterostructured MCs for Alkali Metal−Ion Batteries

### 3.1 Lithium‐Ion Batteries

Since first commercialized by Sony Corporation in 1990, LIBs have shown fast growth and revolutionized the form of consumer electronics.^[1a]^ They possess many clear benefits like high energy density, no memory effect, and negligible self‐discharge. At present, LIBs have been considered as dominant in the power source market, from portable electronic devices to electric cars.^[^ ^15^ ^]^ The working principle of LIBs is based on the shuttle of Li^+^ between the anode and the cathode to provide the electrochemical capacity. As for anode materials of LIBs, graphite is the most applied anode because of its low potential profile and stable cycling performance. Unsatisfactorily, the theoretical capacity for graphite is only of 372 mA h g^−1^.^[^ ^16^ ^]^ Therefore, searching for a new anode with high performance for LIBs is an urgent need. Tremendous studies have reported some alternative anode materials with higher specific capacity, better rate property, and longer life span, including carbonaceous materials with various morphologies,^[^ ^17^ ^]^ Si anode,^[^ ^18^ ^]^ metal oxides,^[^ ^19^ ^]^ alloy anode,^[^ ^20^ ^]^ Ti‐based compounds,^[^ ^21^ ^]^ and lithium metal.^[^ ^22^ ^]^ Therefore, MCs have been widely exploited for LIBs due to their outstanding advantages such as high specific capacity, better structural stability, and ease of preparation with specific morphology. These materials used in LIBs are based on the conversion reaction, which is considered to be able to offer higher reversible capacity. The MCs with different microstructures and morphologies would present unusual performances. Constructing heterostructures in MCs is one of the typical preparation concepts to improve their properties in LIBs. The function of heterojunctions in LIBs mainly is the development of the BIEF effect. This phenomenon‐can efficiently expedite the ion diffusion and charge transportation and further improve the rate performance of bulk MCs.

**Figure 2:**
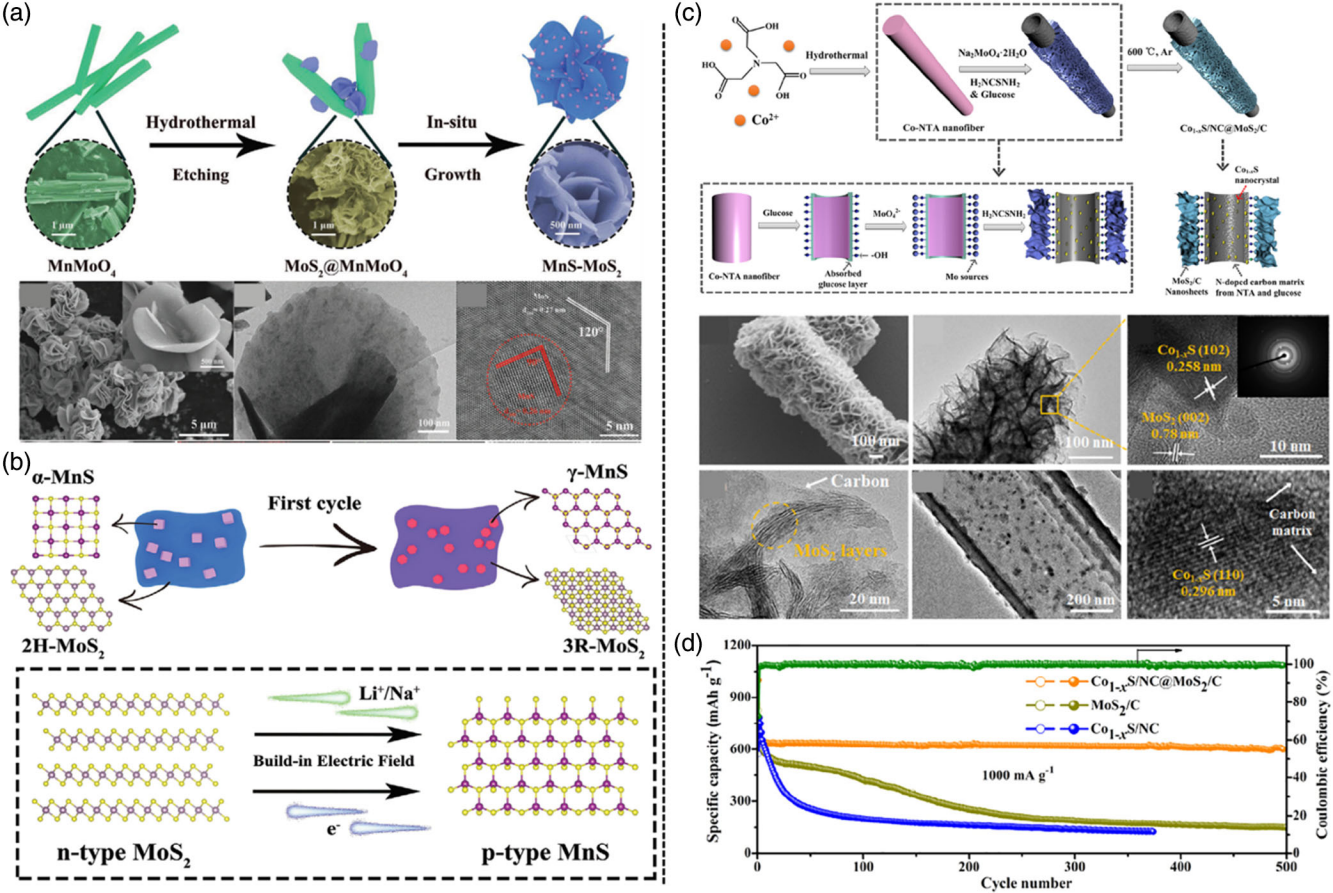
a) Synthesis scheme and morphology of MnS−MoS_2_ heterostructure. b) Mechanism of MnS−MoS_2_ upon the electrochemical procedure: A scheme of phase transition in the first cycle and the BIEF. c) Illustration of the formation strategy of the Co_1–*x*_S/NC@MoS_2_/C hierarchical hollow nanofiber and morphology characterization. d) Electrochemical performance of Co_1–*x*_S/NC@MoS_2_/C for LIBs. a,b) Reproduced with permission.^[^ ^25^ ^]^ Copyright 2020, Wiley‐VCH. c,d) Reproduced with permission.^[^ ^32^ ^]^ Copyright 2020, Elsevier B.V. and Science China Press.

Due to high theoretical capacity of 670 mA h g^−1^, MoS_2_ as a typical MC has gained attention. In general, on the strength of different configurations of Mo and S atoms, MoS_2_ has three kinds of phases: 1 T, 2 H, and 3 R phase. The 1 T phase is a metastable metallic state, whereas the 2 H phase is a stable semiconducting state.^[^ ^23^ ^]^ 3 R phase possesses a special structure that breaks the inversion symmetry, leading to more attractive properties.^[^ ^24^ ^]^ Wu and coworkers^[^ ^25^ ^]^ designed and synthesized an anode with a heterostructure that was constructed by MnS nanoparticles embedded in MoS_2_ nanosheets (**Figure** **2**a,b). MnS is considered as a promising anode material for secondary battery systems due to its lower redox potential and high theoretical capacity of 616 mA h g^−1^.^[^ ^26^ ^]^ MnS also has three different phases: α‐MnS (rock‐salt type), β‐MnS (zinc‐blende type), and γ‐MnS (wurtzite type). Lithium‐ion storage should be significantly enhanced by the phase transition of MoS_2_ and MnS. Likewise, high‐content 1 T‐phase MoS_2_ can elevate the poor electric conductivity and provide more specific capacity.^[^ ^27^ ^]^ MnS undergoes phase transition from α‐MnS to β‐MnS during the conversion reaction that significantly increases Li‐ion storage. In a word, the heterostructure can offer phase transition and BIEF to enhance the Li^+^ diffusion kinetics, improve the charge transport, and suppress the volume change.

In addition, cobalt sulfide is another metal sulfide with high conductivity, whereas its sluggish ion‐diffusion ability results in poor structural stability and terrible electrochemical properties.^[^ ^28^ ^]^ Therefore, reasonable design to combine the advantages of MoS_2_ and CoS into a perfect architecture may lead to surprising effects than two single components.^[^ ^29^ ^]^ Based on density functional theory (DFT) analysis, Ramos et al.^[^ ^30^ ^]^ reported a model for Co_9_S_8_/MoS_2_ heterointerface that shows that the creation of vacancy sites on Mo atoms would interact with Co atoms and that the generation of Co−Mo bonds can induce intense electron transfer between Co and Mo atoms. Inspired by this, Fang and coworkers^[^ ^31^ ^]^ used Co−MOF (ZIF‐67) hollow spheres as template and precursor; then, through the calcination and hydrothermal process, CoS/NC@MoS_2_ hollow nanospheres were prepared. When used as anode for LIBs, these hybrids present high reversible capacity and favorable cycling stability. Zhu and coworkers^[^ ^32^ ^]^ used a hydrothermal method to synthesize 1T−MoS_2_/carbon nanosheet‐decorated Co_1−*x*_S/N‐doped carbon hollow nanofibers (Co_1−*x*_S/NC@MoS_2_/C) by a unique coordination polymer fiber template (Figure 2c,d). The cobalt‐based 1D coordination polymer precursor (Co−NTA) should be transformed in situ into N‐doped carbon hollow fibers embedded with Co_1−*x*_S nanoparticles (Co_1−*x*_S/NC), which can enable the confined growth of MoS_2_ nanoflakes and guarantee homogeneous distribution. Notably, without extra etching, the formation of Co_1−*x*_S/NC hollow fibers and the growth of MoS_2_ could occur simultaneously, which makes the synthetic process speedy and straightforward. This special hierarchical structure of vertically aligned nanosheet‐decorated hollow nanofibers can offer rich active surfaces and short ion‐diffusion distances, which contribute to electron transportation and ion migration.

In view of the aforementioned unique advantages of MnS and CoS, Wu and coworkers^[^ ^33^ ^]^ developed a convenient metal−cyanide coordination polymer‐template approach to fabricate a hollow cube constructed by embedding MnS/Co_1−*x*_S hetero‐nanocrystals within conductive porous carbon@rGO. Namely, Mn^2+^ was first absorbed by electronegative GO sheets and then the Mn_3_[Co(CN)_6_]_2_·9H_2_O Prussian blue analogue was in situ nucleated on GO sheets to achieve precursor. After carbonization and sulfidation of the precursor, the MnS/Co_1−*x*_S@C@rGO composites were obtained at a high temperature. The hierarchically porous hollow architecture, uniformly conductive carbon matrix, and the synergistic effect among the components allow for as‐prepared cube‐like composites with superior electrochemical properties when applied as anodes for LIBs.

Fabrication of multidimensional heterostructure material can also enhance its electrochemical reaction kinetics. For example, Sun et al.^[^ ^34^ ^]^ adopted simultaneous decomposition and sulfidation of a glucose‐coated bi‐MOF template (CH_4_N_2_S@Fe_2_Ni MIL−88) to design a special 0D/1D heterostructured composite of carbon‐encapsulated iron–nickel sulfide nanodots anchored on carbon nanorods (C@FeNi–S NDs/CNR). The as‐prepared nanodot/nanorod heterostructure endows fast ion and electron transport kinetics, inhibits polysulfide dissolution, and guarantees structural integrity during the lithiation/delithiation process. Furthermore, several other configurations with heterostructures have also been reported in LIBs, including starfish‐like Zn_*x*_Co_1−*x*_S@C–CNTs,^[^ ^35^ ^]^ hollow CoS@1T‐MoS_2_,^[^ ^36^ ^]^ mesoporous NiS_2_@CoS_2_@C@C,^[^ ^37^ ^]^ nanocage CoMn−S/rGO,^[^ ^38^ ^]^ FeS_2_/CuS nanospheres,^[^ ^39^ ^]^ and so on.

### 3.2 Sodium‐Ion Batteries

Due to the attractive features of abundant reserve and low costs of sodium resources, SIBs have been identified as one of the most promising alternatives to LIBs for the application of large‐scale energy‐storage devices.^[^ ^40^ ^]^ Because the electrochemical reaction mechanism for SIBs are quite similar to LIBs, many electrode materials with superior Li‐storage performance have been investigated for Na storage by analogy. However, compared with lithium ions, sodium ions exhibit limited characteristics like larger ionic radius, heavier atomic mass, higher reduction potential, and poorer diffusion kinetics, leading to a great challenge for the exploration of sodium‐ion insertion materials with satisfactory electrochemical properties. In the past few years, numerous studies have been focused on potential materials for SIBs, including layered transition metal oxides,^[^ ^41^ ^]^ polyanions,^[^ ^42^ ^]^ Prussian blue,^[^ ^43^ ^]^ carbonaceous materials,^[^ ^44^ ^]^ and so on. Obviously, Na^+^ diffusion needs a larger tunnel structure. Transition MCs possess open layered frameworks, which are deemed promising candidates for SIBs.^[^ ^45^ ^]^

**Figure 3:**
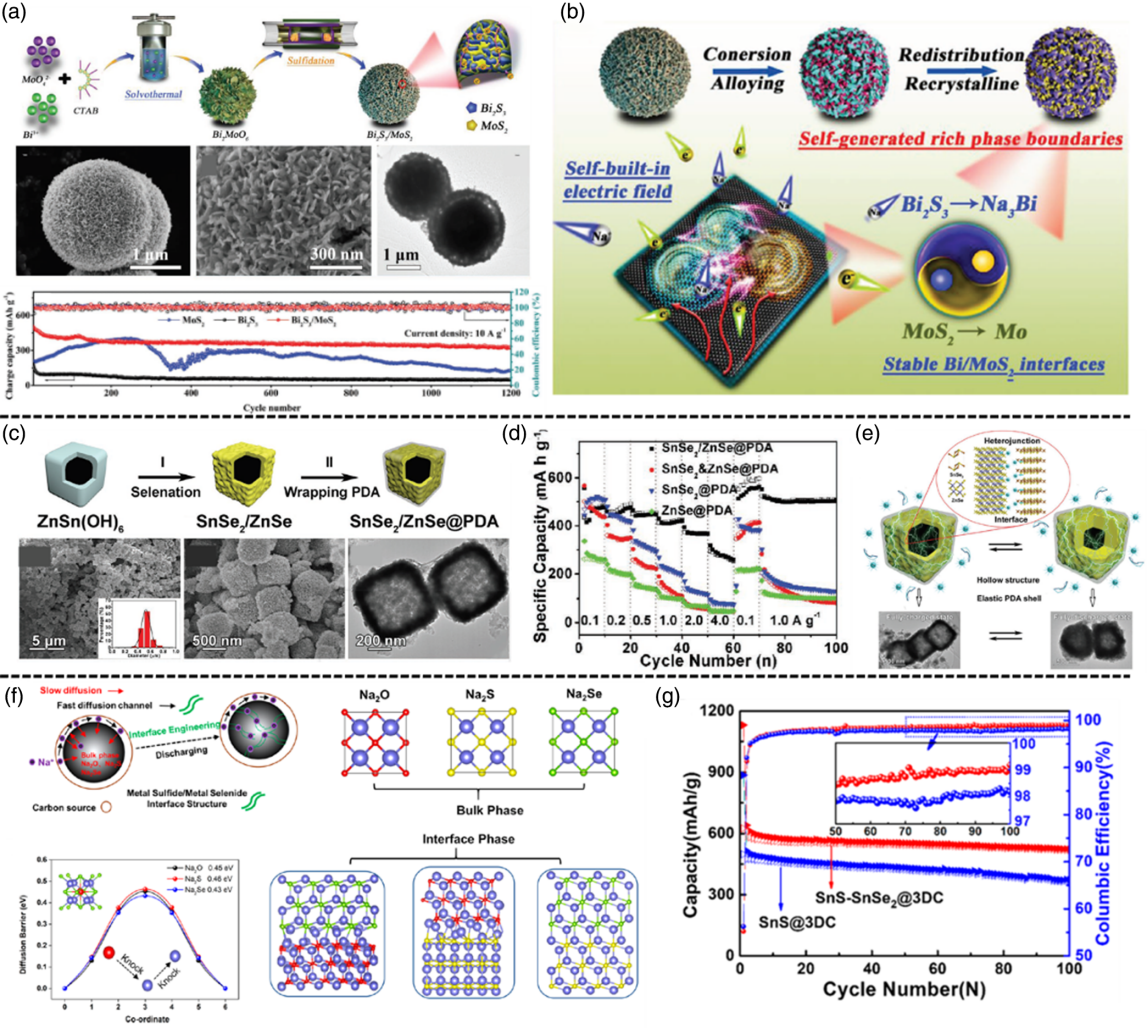
a) The systematic illustration for synthesis process, morphology characterization, and cycle performance of Bi_2_S_3_/MoS_2_ electrode. b) The mechanism scheme of Bi_2_S_3_/MoS_2_ composite with self‐generated phase boundaries. c) Schematic illustration of the preparation, progress, and morphology characterization for SnSe_2_/ZnSe@PDA nanobox. d) Rate capability of SnSe_2_/ZnSe@PDA, SnSe_2_&ZnSe@PDA, ZnSe@PDA, and SnSe_2_@PDA. e) Schematic diagram of changes in SnSe_2_/ZnSe@PDA nanobox during the charge–discharge process. f) The illustration for the interface engineering: The atomic configurations of bulk phase of Na_2_O, Na_2_S, and Na_2_Se; The diffusion energy barrier of Na atom in the bulk phases; The optimized structures of the interface of Na_2_O/Na_2_Se, Na_2_O/Na_2_S, and Na_2_S/Na_2_Se. g) Cycle performance of the SnS@3DC and SnS−SnSe_2_@3DC electrodes. a,b) Reproduced with permission.^[48a]^ Copyright 2020, Wiley‐VCH. c−e) Reproduced with permission.^[^ ^52^ ^]^ Copyright 2020, Wiley‐VCH. f,g) Reproduced with permission.^[^ ^53^ ^]^ Copyright 2020, American Chemical Society.

As a kind of anode material for SIB, alloying‐type metal sulfides (MS_*x*_, M refers to Bi, Sb, Sn) have drawn much attention because of their higher theoretical capacity (Sb_2_S_3_: 954 mAh g^−1^; Bi_2_S_3_: 625 mAh g^−1^; SnS_2_: 1136 mAh g^−1^) and unique intrinsic chemical property.^[^ ^46^ ^]^ Designing a heterostructure containing alloying‐type MS_*x*_ would provide remarkable superiority to SIBs.^[^ ^47^ ^]^ Ou and coworkers reported a series of heterogeneous anode materials based on alloying‐type MS_*x*_ and comprehensively discussed the reaction mechanism.^[^ ^48^ ^]^ The heterostructured (SnCo)S_2_/SG nanocubes were synthesized by coprecipitation, GO wrapping, and sulfidation process, showing an excellent rate performance and structure durability. In addition, in situ X‐ray diffraction (XRD) results show that this composite suffers a six‐stage sodium‐storage mechanism containing three electrochemical reactions of intercalation, conversion, and alloying. The first‐principle DFT calculations indicate that a high concentration of p–n heterojunctions at SnS_2_/CoS_2_ interfaces contributes to the outstanding rate performance. Therefore, they adopted the solvothermal method followed with postsulfidation treatment to successfully prepare a heterostructured Bi_2_S_3_/MoS_2_ microsphere as anode for SIBs (**Figure** **3**a,b). From the in situ XRD, ex situ characterization, and DFT calculations, the Na‐storage mechanism and interphase evolution of Bi_2_S_3_/MoS_2_ anode during discharge/charge process were revealed. It is confirmed that the self‐generation of abundant phase boundaries after recrystallization and redistribution of Bi_2_S_3_/MoS_2_ heterointerface can effectively improve the diffusion kinetics and enhance structure reversibility. They also fabricated hierarchical hollow bimetal sulfides coated with nitrogen‐doped graphene (Sb_2_S_3_@FeS_2_/N‐doped graphene, SFS/C). The heterointerfaces between different binary metal sulfides can induce a BIEF and augment active reaction sites, which can significantly enhance the reversibility and kinetics of electrochemical reaction. The special hollow structure and tightly coated N‐doped graphene matrix can offer extra space to buffer the volume expansion during the cycle process, maintain the stability of bulk structure, and guarantee a high specific capacity.

Recent researches show that selenide‐based MCs exhibit comparatively better electrochemical performances than corresponding sulfides.^[^ ^49^ ^]^ Compared with O and S, metal selenides have a preferable electrical conductivity due to the narrower energy gap. Furthermore, Se possesses a larger radius so that the M−Se bonds are relatively weaker, which could contribute to fast kinetics for the conversion reactions.^[^ ^50^ ^]^ In addition, binary transition MCs are inclined to form a larger crystal size during the synthesis process. In the electrochemical reaction process, although nanomaterial can shorten the diffusion pathway, nanomaterials are usually thermodynamically unstable and tend to agglomerate, which would lead to terrible electrochemical performance.^[2a]^ At that point, compared with monometal counterparts, binary transition MCs may present promising performance due to their higher conductivity, larger microsize, and better electrochemical activities. Hou and coworkers^[^ ^51^ ^]^ used a valid hydrothermal method followed by the selenization and calcination process to synthesize hierarchically porous spheres of iron cobalt binary metal selenide Fe_2_CoSe_4_. The combination of iron and cobalt as a binary metal selenide would make the composite own “metal‐like conductivity,” which is expected for the enhancement of intrinsic conductivity to offer a high rate performance. Jiao and coworkers^[^ ^52^ ^]^ designed a heterojunction SnSe_2_/ZnSe nanobox wrapped by a polydopamine shell (SnSe_2_/ZnSe@PDA), which shows the significant improvement of thermodynamic stability benefiting from the formation of long‐range disorders and lattice distortions inside the material (Figure 3c–e). In addition, the realignment of the electrons around the phase boundary can enhance the adsorption ability and elevate the diffusion kinetics for sodium ions.

Unlike the majority of studies based on bimetal sulfide/selenide, Zhang and coworkers^[^ ^53^ ^]^ reported a systemic investigation of the conspecific metal with dissimilar chalcogenides (Figure 3f,g). They adopted first‐principle calculations to analyze the effect of different heterostructure interfaces for discharging products (Na_2_O, Na_2_S, Na_2_Se) on the rate property, suggesting that the Na_2_S/Na_2_Se interface has the lowest diffusion energy barrier of 0.39 eV for Na among another three kinds of interfaces (Na_2_O/Na_2_S, Na_2_O/Na_2_Se, and Na_2_S/Na_2_Se) because of its smallest recorded interface deformation, similar electronegativity, and lattice constant. Further, their experimental results further confirm that the hierarchical metal sulfide/metal selenide (SnS/SnSe_2_) shows excellent rate performance. The proposed design strategy would shed light on constructing a series of conspecific metal with dissimilar chalcogenides with high rate property for other battery systems. In addition, several other configurations with heterostructures have also been reported in SIBs, including mesoporous NiS_2_@CoS_2_@C@C,^[^ ^37^ ^]^ (CoFe)Se_2_@CNS^[^ ^54^ ^]^ (CNS, carbon nanofiber skeleton), hierarchical NiS/FeS−CNT^[^ ^55^ ^]^ (CNT, carbon nanotube), SnS_2_/NiS_2_ hetero‐nanosheet arrays,^[^ ^56^ ^]^ CoSe_2_/(NiCo)Se_2_ box‐in‐box hollow nanocubes,^[^ ^57^ ^]^ and so on.

### 3.3 Potassium‐Ion Batteries

As alternatives for LIBs, other alkali ion batteries such as KIBs are also gaining considerable interest in these years due to abundant potassium sources.^[^ ^58^ ^]^ Compared with SIBs, the standard redox potential of KIBs is −2.93 V, which is lower than that of SIBs (−2.71 V for Na/Na^+^) and close to that of LIBs (−3.04 V for Li/Li^+^). The lower standard potential makes KIBs operate under a wider operating voltage window and exhibit a higher energy density, realizing KIBs as a suitable candidate for next‐generation rechargeable batteries.^[^ ^59^ ^]^ However, the large radius of potassium ion (1.38 Å) fails to achieve fast insertion/extraction kinetics within anodes, leading to serious volume change and poor electrochemical performance. Therefore, it is still arduous to explore an appropriate and efficient anode material for KIBs with high specific capacity, smooth reaction kinetics, and long cycle life.

In the past few years, many papers have reported carbonaceous materials,^[^ ^60^ ^]^ alloying materials,^[^ ^61^ ^]^ and other materials^[^ ^62^ ^]^ as anodes for KIBs. Recently, metal sulfides have shown huge potential as potassium‐storage materials, which is mainly due to their high theoretical capacities.^[^ ^63^ ^]^ As widely explored in LIBs and SIBs, layered‐structure MoS_2_ can also be used in KIBs because of its expanded interlayer spacing (0.615 nm), which is favorable for fast K^+^ diffusion. Unfortunately, it has some shortcomings like poor conductivity and unsatisfied structural stability.^[^ ^62^ ^]^ An alternative method is to introduce heterophases into MoS_2_ bulk to elevate charge transport for enhanced electrochemical reaction kinetics. Liu and coworkers^[^ ^64^ ^]^ prepared an MoS_2_‐based heteromaterial with a conductive Fe_9_S_10_ core and carbon coating (Fe_9_S_10_@MoS_2_@C) as anode for KIBs. Therefore, the Fe_9_S_10_ core may significantly improve the electronic conductivity, facilitate the densification of MoS_2_ particles, and construct rich heterointerfaces with a strong BIEF, which achieves high energy density and increased reaction kinetics. Moreover, experimental results and DFT calculations suggest that the generation of heterointerfaces contributes to the improvement of fast charging capability and cycle span by reducing the ion‐diffusion energy barrier and strengthening geometry architecture.

**Figure 4:**
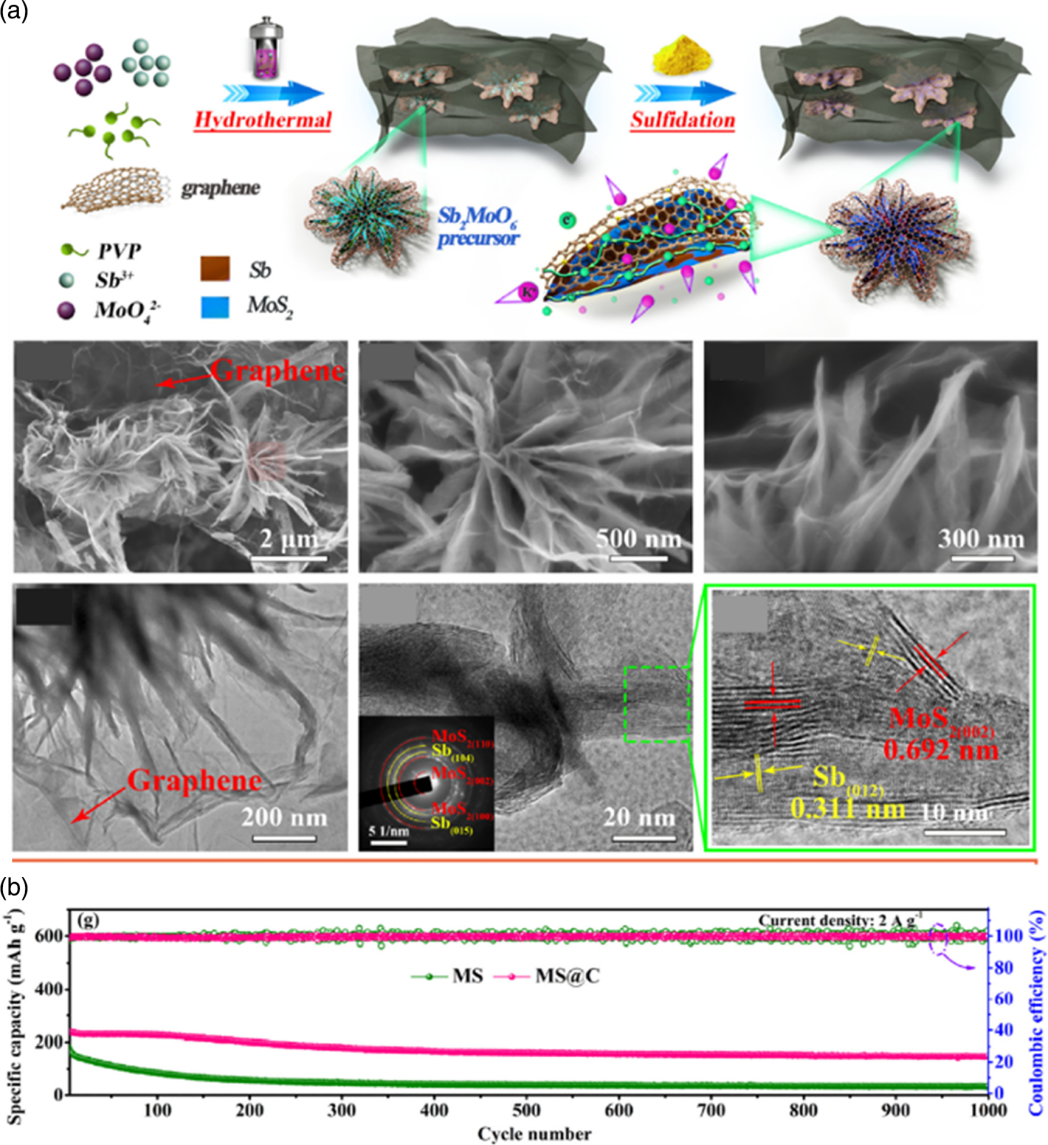
a) The systematic illustration for synthesis process and morphology characterization of MoS_2_/Sb@C electrode. b) The cycle performance of MoS_2_/Sb@C electrode. a,b) Reproduced with permission.^[^ ^66^ ^]^ Copyright 2020, Elsevier B.V.

Except for being equipped with metal sulfides as heterophases, other compounds including metal oxides or alloying material should also be used to build heterostructures to modify the performance of MoS_2_. Wang and coworkers^[^ ^65^ ^]^ design and fabricate three‐layered heteroanode materials for KIBs with a core−shell structure, which is composed of porous metallic TiNb_2_O_6_ as the core and carbon‐coated MoS_2_ nanosheets as the shell (TiNb_2_O_6_@MoS_2_/C). TiNb_2_O_6_ with unsaturated oxygen atoms may exhibit a metallic conducting behavior, greatly reinforcing the internally electronic kinetics of the composite. In addition, the porous TiNb_2_O_6_ microspheres can offer extra capacity, restrain the agglomeration of MoS_2_ nanosheets, increase the contact area between the active material and electrolyte, and suppress the volume expansion of active materials. In addition, the rough surface of TiNb_2_O_6_ microspheres would offer high‐energy nucleation sites for the dense growth of ultrathin MoS_2_, which effectively increases the cohesion of the heterogeneous layers and thus demonstrates a high structural stability. As for hybridizing with alloy‐type materials, MoS_2_/Sb coated by a N‐doped graphene network with a hierarchical chrysanthemum‐like heterostructure as anode material for PIBs was rationally synthesized (**Figure** **4**).^[^ ^66^ ^]^ When used as anode for KIBs, the metallic Sb presents high theoretical capacity of 660 mAh g^−1^ and low‐voltage plateau. However, the inevitable volume variation during the repeated K^+^ (de)intercalation processes would result in the serious electrode pulverization and disastrous structural collapse. The heterointerfaces between MoS_2_ and Sb nanoparticles can confine the huge volume expansion and prevent the crystal coarsening induced by the phase transformation, which efficiently inhibits the structural damage. More importantly, the synergistic effects induced by the 3D integral conductive networks consist of an exterior N‐doped graphene nanosheet, and the interior MoS_2_/Sb heterostructure can significantly intensify the reaction kinetics and exhibit an ultrahigh rate performance.

## 4 Heterostructured MCs for Li−S Batteries

The Li−S batteries are considered as one of the promising alternatives to LIBs due to their high theoretical cathode capacity of 1672 mAh g^−1^ and high theoretical energy density of 2600 Wh kg^−1^. Meanwhile, the abundant reserves and low costs of sulfur make Li−S batteries applicable for commercialization. Nevertheless, the practical application of Li−S batteries is impeded due to the poor cycling performance and rate capability.^[^ ^67^ ^]^ The soluble intermediates, lithium polysulfides (LiPSs), produced during discharge and charge processes can trigger the shuttle effects, leading to continuous sulfur loss, electrolyte consumption, and lithium anode corrosion. In addition, the active material sulfur and discharge products (Li_2_S_2_/Li_2_S) are electrically insulating, which induces sluggish redox kinetics and severe cathode surface passivation, resulting in large polarization and low sulfur utilization.

To alleviate these issues, research efforts have been put into designing the host for sulfur cathode. Nonpolar carbon materials, such as carbon nanotubes, graphene, and porous carbon with high conductivity and large surface area, effectively enhance the electronic conductivity of the electrode and meanwhile accommodate the volume change of sulfur during discharge−charge processes. However, the interaction between polysulfides and carbon materials is insufficient to anchor the polysulfides and suppress the shuttle effects, and the conversion of polysulfides on carbon materials is always sluggish. Therefore, transition MCs have been extensively investigated as sulfur hosts for Li−S batteries. MCs can not only chemically trap the polysulfides, but also enhance the polysulfides’ conversion due to their electrocatalytic capability.

**Figure 5:**
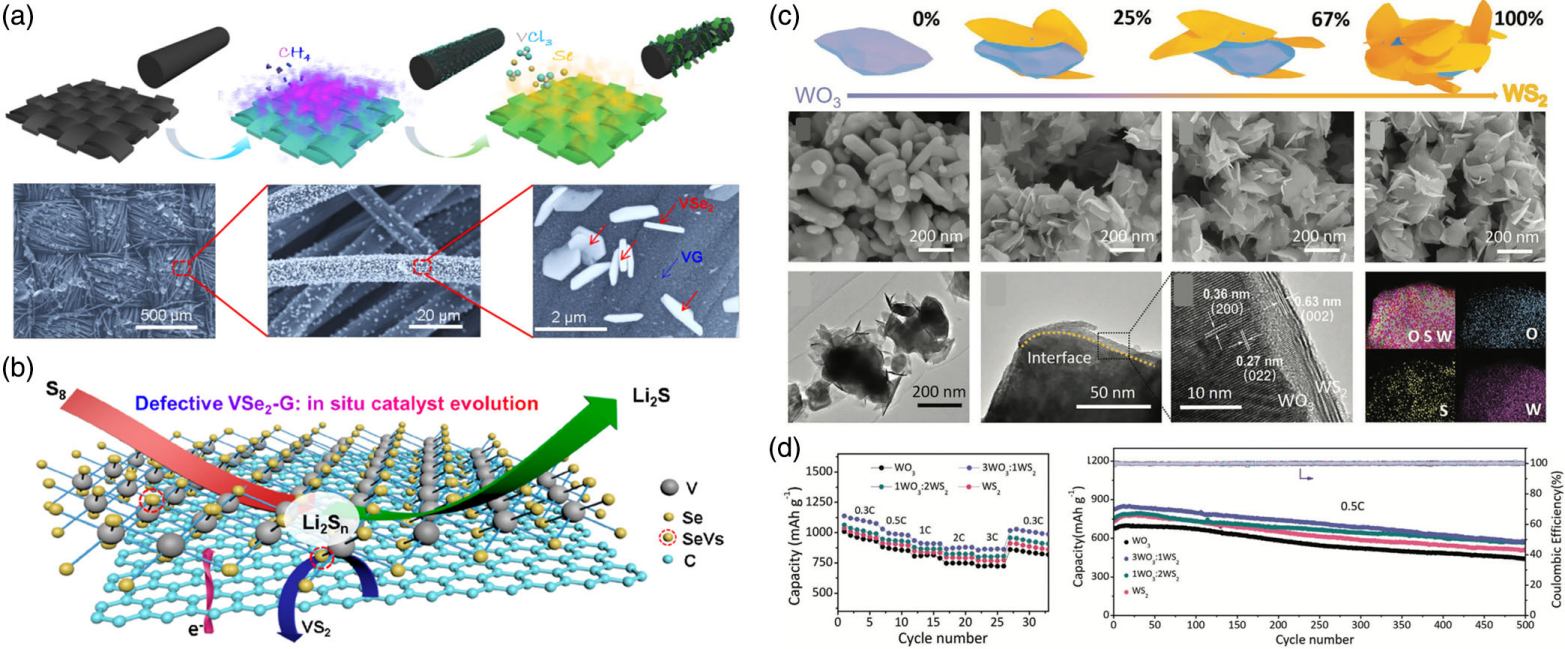
a) Schematic illustration of the all‐CVD growth synthetic process of VSe_2_−VG heterostructure material on CC. b) Scheme showing the discharge process at the SeVs−VSe_2_−VG@CC/S cathode, where the defective VSe_2_−graphene heterostructures not only strengthen the chemical adsorption to polysulfides but also improve the catalytic ability and facilitate the electron/ion conductivities in the host matrix. c) The structure and morphology changes of WS_2_–WO_3_ heterostructures with different sulfurization degrees and d) their corresponding electrochemical performance as the additive (5 wt%) to sulfur cathode for Li−S batteries. a,b) Reproduced with permission.^[69a]^ Copyright 2020, American Chemical Society. c,d) Reproduced with permission.^[71a]^ Copyright 2020, Wiley‐VCH.

To highly utilize the advantages of MCs, the MC/graphene heterostructures are constructed and applied for Li−S batteries. A freestanding and 3D graphene/1 T MoS_2_ (3DG/TM) heterostructure was designed, which possesses great electrocatalytic capability for lithium polysulfides.^[^ ^68^ ^]^ The graphene/1 T MoS_2_ heterostructure was built via few‐layered graphene nanosheets sandwiched by few‐layered 1 T MoS_2_ nanosheets, which is in favor of electrolyte penetration and Li‐ion transfer. Both the graphene and 1 T MoS_2_ nanosheets show high conductivities, which facilitate electron transfer. The advantages of the heterostructure are high electrocatalytic efficiency for polysulfides, due to the presence of sufficient electrocatalytic active sites and excellent ion/electron transfer. To conquer the issues of the uniformity and poor tailorability in heterostructures constructed via the normally used wet chemistry method, the CVD approach was used to grow MC/graphene heterostructures on target substrates, thus constructing the heterostructures with superior control for surface coverage and crystal quality.^[^ ^69^ ^]^ The defective VSe_2_ and vertically oriented graphene (VG) nanosheet were combined and in situ built on a carbon cloth (CC) to form the sulfiphilic heterostructure.^[69a]^ The VSe_2_−VG heterostructure works as an efficient LiPS electrocatalyst to propel polysulfide conversion (**Figure** **5**a,b).

In addition to the MC/graphene heterostructures, the MC/TMC,^[^ ^70^ ^]^ MC/metal oxides,^[^ ^12^, ^71^ ^]^ MC/LDH,^[^ ^72^ ^]^ and MC/MXene^[^ ^73^ ^]^ heterostructures are also designed and constructed for Li−S batteries. When applied for Li−S batteries, the MC in these heterostructures possesses good electrocatalytic capability for polysulfides conversion, and the metal oxides or MXenes show good trapping ability with polysulfides. Thus, the heterostructures could synergistically enhance the trapping, diffusion, and conversion of polysulfides and improve the electrochemical performance of Li−S batteries.[^71^, ^73^] For instance, WS_2_ nanosheets–WO_3_ nanoparticle heterostructures were prepared by the vapor sulfurization of WO_3_ nanoparticles (NPs).^[71a]^ The interfacial contact between WS_2_ and WO_3_ is tight and they are atomically matched. Ultimately, abundant active interfacial active sites are formed in the WS_2_–WO_3_ heterostructure. Meanwhile, the ratio of WS_2_ and WO_3_ could be regulated to effectively alter the performance of this electrocatalytic heterostructure (Figure 5c,d).

## 5 Heterostructured MCs for Metal−Air Batteries

### 5.1 Li−O_2_ Batteries

Among the next‐generation alternatives beyond LIBs, in addition to the Li−S batteries, the rechargeable Li−O_2_ batteries have attracted tremendous interests due to their high theoretical energy density up to 3500 Wh kg^−1^. The typical electrochemical reactions in the Li−O_2_ batteries are the reversible redox reactions between lithium ions and oxygen, namely, O_2_ + 2Li^+^ + 2e^−^ ⇌ Li_2_O_2_. However, the Li−O_2_ batteries are still far from practical applications, which results from the inherent insulating and insoluble properties of the discharge product (Li_2_O_2_) in the aprotic electrolyte. During the discharge process (ORR), Li_2_O_2_ precipitations block the transport channels of reactants and passivate the cathode surfaces, leading to the deterioration of electrical contact between effective active species and catalysts, which finally increases the charge transfer impedance. In contrast, during the charge process (OER), the insulating Li_2_O_2_ precipitations are not easy to decompose, delivering slow reaction kinetics and high overpotential. Due to these problems, the Li−O_2_ batteries show unsatisfactory electrochemical performance with poor rate capability, low discharge/charge capacity, and limited cycling life.

To enhance the electrochemical kinetics of oxygen redox reactions in Li−O_2_ batteries, it is necessary to develop bifunctional electrocatalysts for ORR and OER.^[^ ^74^ ^]^ A variety of electrocatalysts such as noble metals, metal oxides, and MCs have been extensively reported.^[^ ^75^ ^]^ Among them, transition MCs are regarded as the most promising electrocatalysts for Li−O_2_ batteries, due to their alterable bandgaps, tunable surface chemistry, and abundant edge and plane active sites.

**Figure 6:**
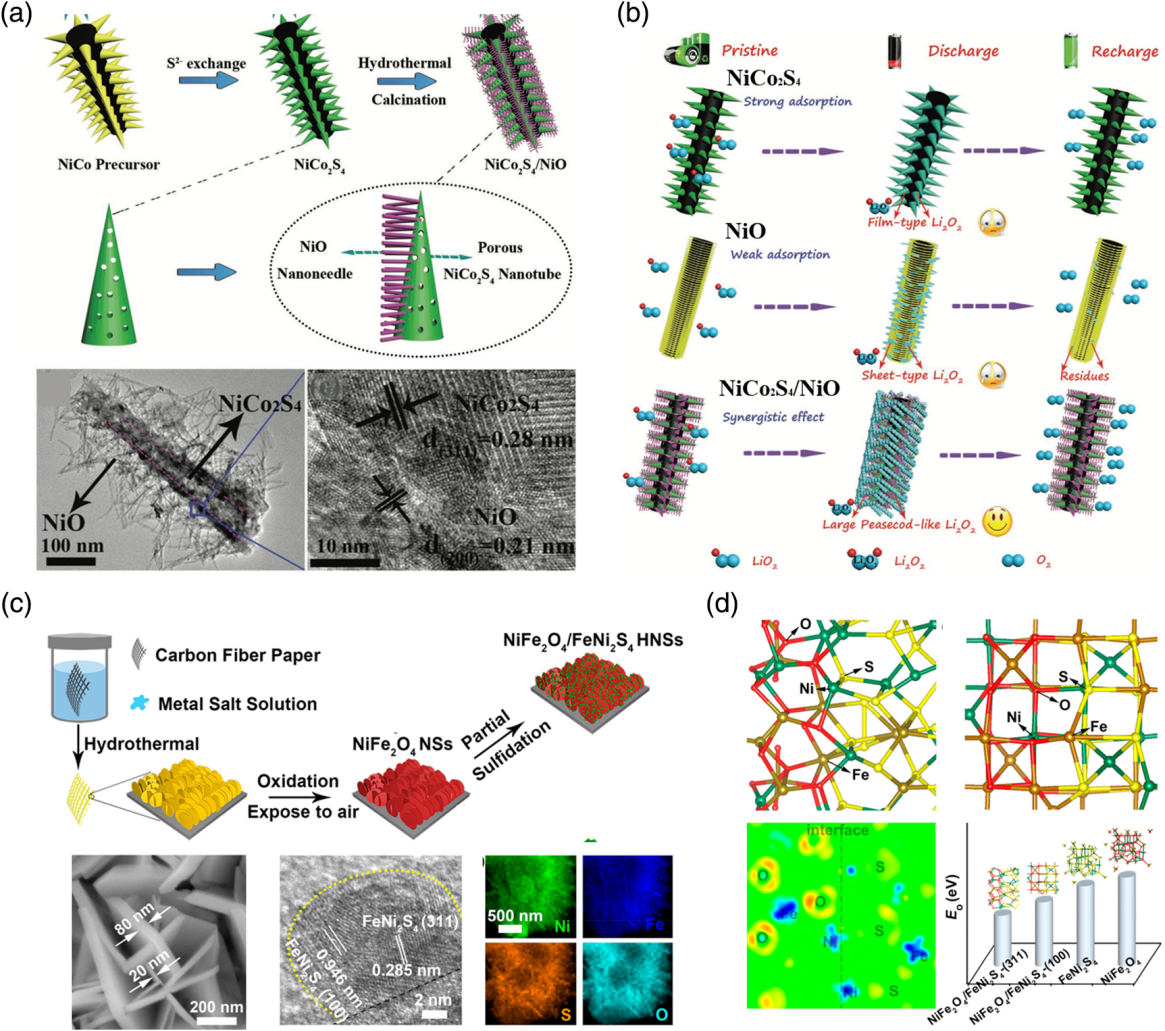
a) The preparation process of NiCo_2_S_4_/NiO heterostructure arrays on carbon paper and the transmission electron microscopy (TEM) images of the NiCo_2_S_4_/NiO heterostructure. b) Schematic illustrations of the working mechanism for the NiCo_2_S_4_, NiO, and NiCo_2_S_4_/NiO electrodes for Li−O_2_ batteries. c) The preparation process and the morphology of NiFe_2_O_4_/FeNi_2_S_4_ heterostructured nanosheets (HNSs). d) DFT calculations to evaluate the surface energetics of different NiFe_2_O_4_/FeNi_2_S_4_ interfaces to study interface−catalysis correlation in this heterostructured catalyst with oxygen electrocatalytic properties. a,b) Reproduced with permission.^[^ ^76^ ^]^ Copyright 2019, Wiley‐VCH. c,d) Reproduced with permission.^[^ ^84^ ^]^ Copyright 2018, American Chemical Society.

To meet the requirements for Li−O_2_ batteries and develop highly efficient bifunctional electrocatalysts, it is very important to construct MC‐based heterostructures with unique morphology. Based on the idea, a 3D hierarchical core−shell heterostructure, with 1D NiCo_2_S_4_ porous nanotubes as the core and 1D NiO nanoneedles as the shell, was constructed on carbon paper (**Figure** **6**a,b).^[^ ^76^ ^]^ The formed array network provides multidimensional charge diffusion pathways and sufficient specific area for discharge products deposition. Moreover, due to the BIEF effect at the NiCo_2_S_4_/NiO heterostructure interface, the interfacial charge transport kinetics is effectively enhanced within the hybrid cathode. Other MC/metal oxide heterostructure catalysts have also been reported.^[^ ^77^ ^]^

Similar to the MC/metal oxide, the MC/MC heterostructures have been reported to improve the electrochemical performance of Li−O_2_ batteries.^[^ ^78^ ^]^ The heterostructure always possesses active interfaces and rich reaction sites. Due to these chemical synergistic effects between the different MCs in the heterostructure, the composite catalyst will exhibit an improved catalytic activity. In addition, the hybrid material with coupled two phases will induce a BIEF, which significantly increases the charge transfer rate during the catalytic processes.

### 5.2 Zn−Air Batteries

Rechargeable zinc−air batteries are considered as the promising candidates to meet our demands for powering electrical devices, due to their high theoretical energy density and low cost. The Zn−air batteries have three main units, including air cathode, alkaline electrolyte, and zinc anode. During the discharge process, the oxygen diffuses into the air cathode and is reduced on the catalyst/cathode interface and forms hydroxide ions in the alkaline electrolyte. During the charge process, oxygen will be produced and diffuses out of the air cathode. Therefore, an air cathode with dual catalytic activity, namely, ORR and OER, should be achieved for the Zn−air batteries.^[^ ^79^ ^]^ Similar to the roles of MC‐based heterostructure catalyst in Li−O_2_ batteries, these materials can effectively promote the ORR and OER reactions in Zn−air batteries, such as NiSe_2_/CoSe_2_,^[^ ^80^ ^]^ Co_9_S_8_/MoS_2_,^[^ ^81^ ^]^ NiFe_2_O_4_/Ni_3_S_4_,^[^ ^82^ ^]^ and NiCo_2_Se_4_/NiCoS_4_.^[^ ^83^ ^]^ The MC‐based hybrid catalysts show great performance in reversible oxygen conversion, and high efficiency, low overpotential, and prolonged cycling life are achieved for rechargeable Zn–air batteries.

Apart from the conventional Zn−air batteries that use the alkline electrolyte, the MC‐based heterostructure also shows good performance in the batteries with a neutral‐pH electrolyte. The neutral electrolyte always possesses extremely low OH^−^ concentration and decreased ionic conductivity; therefore, the main electrochemical reactions (ORR and OER) at the air cathode are expected to be much more kinetically sluggish in neutral electrolyte. To maximize the performance‐neutral Zn–air batteries, NiFe_2_O_4_/FeNi_2_S_4_ heterostructure‐based nanosheets were fabricated, which can promote OER and ORR reactions efficiently, so as to enable neutral Zn–air batteries.^[^ ^84^ ^]^ On the formed oxide/sulfide interfaces, binding energy between the catalyst surface and oxygenated species can be improved to accelerate the kinetics for the main electrochemical reactions (Figure 6c,d).

## 6 Heterostructured MCs for Multivalent Ion Batteries

Commercial LIBs possess decent energy‐storage capacity, but scarce reserves of lithium metal resource and risk of safety due to flammability of nonaqueous electrolytes make it unsuitable for large‐scale energy‐storage grids. In consideration of safety and cost, multivalent ion batteries have successively attracted much attention, including magnesium rechargeable batteries (MRBs),^[^ ^85^ ^]^ zinc‐ion batteries,^[^ ^86^ ^]^ calcium‐ion batteries,^[^ ^87^ ^]^ aluminum‐ion batteries,^[^ ^88^ ^]^ and so on.^[^ ^89^ ^]^ Multivalent metals can not only substitute lithium metal in rocking‐chair cells but also can settle in the aqueous media due to their more stable property of reactivity, even though under the narrow electrochemical windows. In addition, multivalent ions batteries may exhibit elegant capacity and high energy density, benefiting from the multivalent chemistry in the charge−discharge process.^[^ ^90^ ^]^ According to the present situation, the great issue for multivalent ion batteries is still the limited electrochemical compatibility of the electrode materials with the whole battery system. Regarding the inserted cathode materials used in multivalent ions batteries, the repulsive force between electrode materials and shuttling ions is unexpectedly very large and due to that the two or three charge transfers may produce much stronger polarization than that of LIBs. It would result in severe irreversible capacity, sluggish ion‐diffusion kinetics, and inferior rate performance. Therefore, the discovery of applicable cathode materials is the key to improve the electrochemical performance of multivalent ion batteries.

### 6.1 Magnesium Rechargeable Batteries

**Figure 7:**
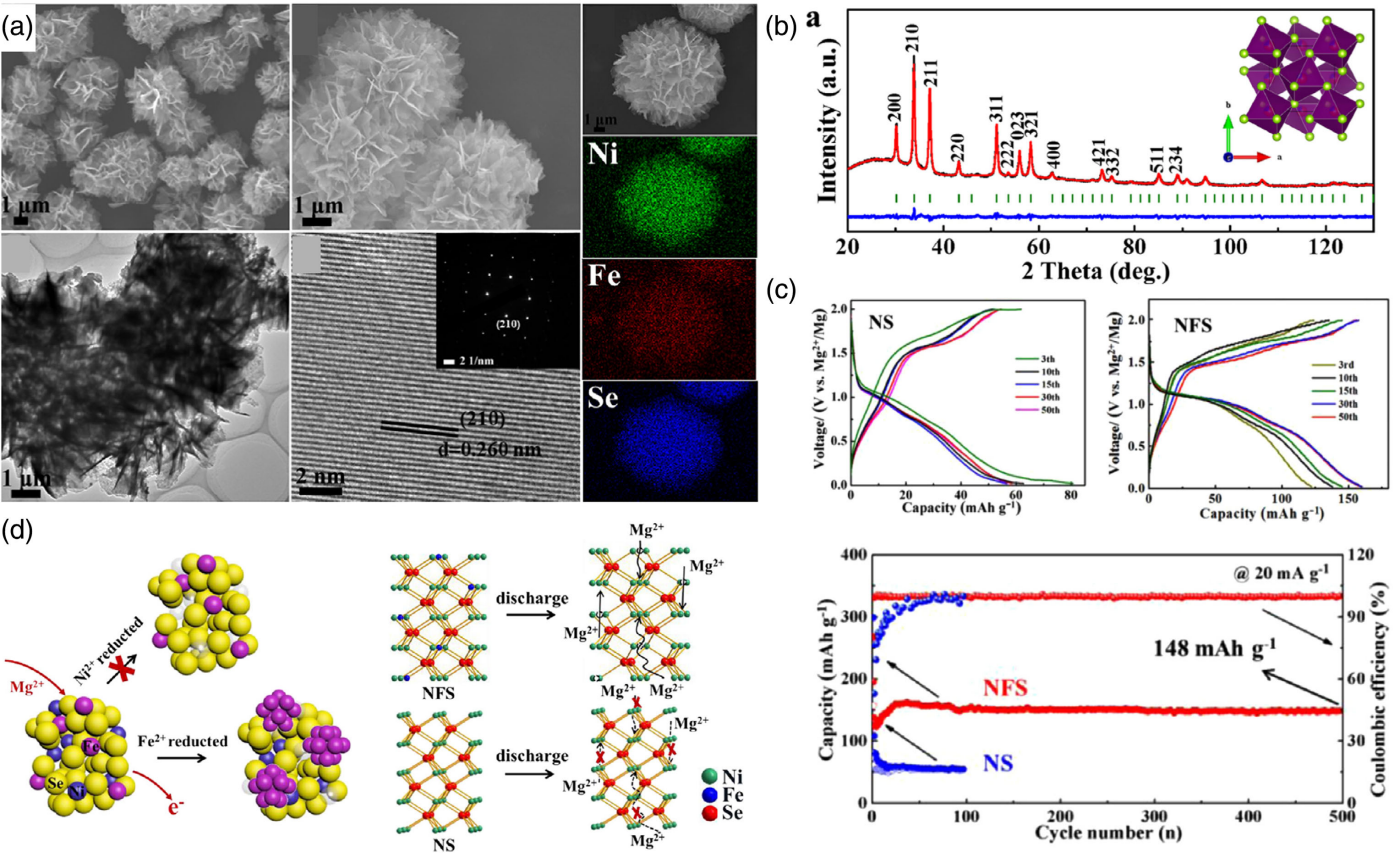
a) Morphology characterization of the as‐prepared Ni_0.75_Fe_0.25_Se_2_. b) Rietveld refined XRD pattern of the as‐prepared Ni_0.75_Fe_0.25_Se_2_. c) Charge/discharge curves of NS (NiSe_2_) and NFS (Ni_0.75_Fe_0.25_Se_2_) at 20 mA g^−1^ and at the 3rd, 10th, 15th, 30th, 50th cycle. Cycling performance at 20 mA g^−1^ of the NiSe_2_ and Ni_0.75_Fe_0.25_Se_2_ electrode tested at room temperature (25 °C) for magnesium batteries. d) Schematic illustration of reaction order of Ni and Fe in the Ni_0.75_Fe_0.25_Se_2_ electrode (blue ball is Ni ions, purple ball is Fe ions, yellow ball is Se ions, and purple cluster is elemental iron). Comparison of increased redox sites of Ni−Fe bimetallic selenide at steady state with pure NiSe_2_. a–d) Reproduced with permission.^[^ ^94^ ^]^ Copyright 2018, Elsevier Ltd.

The prototype system for MRBs was first reported by Aurbach et al., comprising Mg_*x*_Mo_3_S_4_ cathodes with the capacity of 110 mAh g^−1^ and the electrolyte based on Mg organohaloaluminate salts.^[^ ^91^ ^]^ Recent efforts have mainly been devoted to suitable alternative‐like oxides or alloys to improve the electrochemical performance.^[^ ^92^ ^]^ MCs have been studied to present good performance for magnesium batteries because of the weak interactions between Mg^2+^ and soft anion lattice. Although MCs with heterostructure have not been widely synthesized and used in MRBs, bimetallic chalcogenides were explored by some researchers, which may also give us some inspiration.^[^ ^93^ ^]^ Mai and coworkers^[^ ^94^ ^]^ designed the microflower‐like Ni−Fe bimetallic diselenides (Ni_0.75_Fe_0.25_Se_2_) via a facile solvothermal method, showing enhanced diffusion kinetics when used as cathode material for magnesium batteries (**Figure** **7**a,b). At a current density of 20 mA g^−1^, this composite shows an excellent discharge capacity of 190 mAh g^−1^ and can retain the capacity of 148 mAh g^−1^ after 500 cycles (Figure 7c). The excellent electrochemical performance is put down to the bimetallic effects, which can lead to faster Mg ion‐diffusion dynamics and induce more redox sites, compared with unary metal selenide NiSe_2_ (Figure 7d).

To circumvent the limitation of lacking cathode materials in MRBs, recently, hybrid Mg^2+^/Li^+^ batteries (MLIBs) became attractive energy‐storage devices, which simultaneously combine the fast Li^+^‐storage cathode, dendrite‐free Mg anode, and stable Mg^2+^/Li^+^ hybrid electrolyte. Zhang and coworkers^[^ ^95^ ^]^ prepared free‐standing PPy‐encapsulated CoS_2_ nanosheets anchored on the TiO_2−*x*_ nanorod array (TiO_2−*x*_@CoS_2_@PPy) as cathode for hybrid MLIBs. The TiO_2−*x*_ support and stable Ti—S bonds can effectively suppress the dissolution of polysulfide intermediates and inhibit the exfoliation of active material CoS_2_, which demonstrate an outstanding structural stability during cycling. The theoretical calculations suggest that, compared with pristine TiO_2_, TiO_2−*x*_ possesses stronger chemical interactions with CoS_2_, polysulfide intermediates, and the corresponding discharge products.

### 6.2 Aluminum‐Ion Batteries

If a three‐electron‐transfer electrode reaction would be successfully stimulated, Al metal could offer an excellent gravimetric capacity of 2980 mAh g^−1^.^[^ ^88^ ^]^ In addition, considering the abundant aluminum resource, high safety, and nontoxicity, rechargeable aluminum batteries (RABs) are regarded as one of the most promising devices for energy storage.^[^ ^96^ ^]^ However, trivalent Al^3+^ ineluctably suffers severe kinetic issues in cathode materials due to strong electrostatic interactions. Except for carbonaceous‐based material, transition metal sulfides have gradually attracted attention due to their larger interlayer spacings. Most studies focused on monotypic sulfides,^[^ ^97^ ^]^ presenting poor electronic conductivity and inferior ion‐diffusion kinetics. Multicomponent sulfides, by contrast, are expected to exhibit outstanding electronic conductivity and extraordinary electrochemical performance.^[^ ^98^ ^]^

Gao and coworkers^[^ ^99^ ^]^ reported a binary metal sulfide prepared by a strategy combining the layered double hydroxides (LDHs) with graphene oxide (GO) and subsequent sulfidation. At the current density of 1 A g^−1^, the resulting composite (S‐NiCo@rGO) shows a high discharge capacity of 248.2 mA h g^−1^ and maintains a discharge capacity of 83 mA h g^−1^ with nearly 100 % Coulombic efficiency after 100 cycles. The mechanism of this material should be due to the substitution of Ni^2+^ and Co^2+^/Co^3+^ by Al^3+^. Zhang and coworkers^[^ ^100^ ^]^ adopted a hydrothermal method to synthesize Bi_2_S_3_/MoS_2_ nanorod, which is used as a new‐type cathode material for rechargeable RABs, and it demonstrates a specific discharge capacity of 132.9 mA h g^−1^ at a current density of 1 A g^−1^ after 100 cycles (Figure 7c,d). The well‐defined heterojunction can effectively maintain the structural stability of Bi_2_S_3_ along with long‐term cycling, which provides a novel strategy for designing high‐performance cathode materials for RABs.

## 7 Conclusion and Outlook

In summary, this project comments on and reviews the recent developments of various MCs with heterostructures used in four typical categories of rechargeable batteries, including alkali metal−ion batteries, Li−S batteries, metal−air batteries, as well as the multivalent ion batteries. The design and preparation of MCs with heterostructures for rechargeable batteries are also discussed. The significance of the design concept for heterostructures on their electrochemical performances is comprehensively discussed, which includes BIEF, lattice vacancy effect, synergistic effect, and electrocatalysis effect. In fact, one or more of the four advantages may exist in different battery systems, and they mutually reinforce the properties with each other. The heterointerfaces can induce a strong BIEF to accelerate electron and ion diffusion and offer an improved rate performance. Meanwhile, the lattice vacancies produced in phase boundaries would cause aggregation of located electrons to increase electronic conductivity and strengthen electrochemical reaction kinetics. In addition, the MCs‐based heterostructures always possess rich reaction sites and active interfaces for ORR/OER, which can be the promising electrocatalysts for metal−air batteries. Moreover, incorporation of dissimilar components will contribute to enhancing the structural stability because of the formation of stronger chemical bonds and interfacial protective layer, leading to better cycle performance.

Although numerous promising heterostructured MCs with encouraging properties have been reported, shortages and challenges still exist especially in practical applications. 1) Design Thought: Among enormous quantity of MCs, how to choose two or more to combine them into one compound, how to make sure of the compatibility of two different components, and how to prepare the optimal product with proper ratio remain the main issues. Theoretical calculation and material simulation technology play more important roles in the research and designing processes of advanced material fields, which should be effectively borrowed to construct the heterostructured MCs. 2) Synthesis Cost: Commonly, the preparation technologies and synthetic routes for fabricating heterostructured MCs are more complicated, most of them require multistep reactions. Therefore, developing versatile, high‐efficiency, and low‐cost methods to prepare satisfied heterostructured MC electrodes is also a difficult problem that must be faced. 3) Mechanism Analysis: Heterostructured MC is a composite containing various component and diversified heterointerfaces, resulting in extremely complicated electrochemical reactions. Although part of the studies described possible energy‐storage mechanisms based on characterization, it is not convincing and fails to clarify the multiple mechanisms in detail. Thus, the structure−function relationships between heterostructures and outstanding electrochemical performance should be settled urgently by the classical electrochemical analysis techniques and advanced in situ characterization techniques. 4) Compatibility: At present, the studies of heterostructured MCs are still in the basic research stage and most of them mainly focus on the half cells. Their compatibilities with counter electrodes, electrolytes, and separators when applied in full cells should be noted, which directly decide the possibility of the practical application for high‐performance battery systems. Namely, we should hammer at four aspects of the development direction for heterojunctions applied in rechargeable batteries, which include theoretical computational simulation, synthesis cost reduction, state‐of‐the‐art characterization techniques, and ponch cell assembling.

Finally, with the rapid development of nanomaterial science, material genomics technology, and in situ characterization technique, heterosturctured MCs or other heterosturctured material may exhibit better performance and even be extended to other unused battery systems like calcium rechargeable batteries and iron rechargeable batteries. For different types of batteries, in addition to structure and performance, other important issues should also be carefully considered for the design of heterostructure chalcogenide materials, including the compatibility among the key materials and the interface characteristics between the electrode and the electrolyte. Due to their unique structural and compositional features, it is expected that the design concept of heterostructures will strut their stuff in the whole energy‐storage applications.

## Conflict of Interest

The authors declare no conflict of interest.
